# Cutaneous tuberculosis: epidemiological, clinical, diagnostic and therapeutic update^☆^

**DOI:** 10.1016/j.abd.2021.07.004

**Published:** 2022-01-04

**Authors:** Arival Cardoso de Brito, Clivia Maria Moraes de Oliveira, Deborah Aben-Athar Unger, Maraya de Jesus Semblano Bittencourt

**Affiliations:** Dermatology Service, Universidade Federal do Pará, Belém, PA, Brazil

**Keywords:** Epidemiology, *Mycobacterium tuberculosis*, Pathogenesis, homeopathic, Tuberculosis, Tuberculosis, cutaneous

## Abstract

Tuberculosis is certainly one of the diseases considered to be ancient on planet Earth. The etiological agent of tuberculosis is *Mycobacterium tuberculosis*. This terrible bacterial infection still results in severe socioeconomic consequences to date, and its complete eradication represents a great challenge. It constitutes one of the most important public health problems in developing countries. According to the World Health Organization, this infection results in more than 4,000 deaths daily worldwide, with 10.4 million being affected annually and 1.5 million deaths from TB every year. With the emergence of the HIV/AIDS pandemic, the disease became the main cause of morbidity and mortality in patients infected with the human immunodeficiency virus. Cutaneous tuberculosis is a rare infection that represents 1% to 1.5% of extrapulmonary tuberculosis, whose etiological agents are *Mycobacterium tuberculosis*, *Mycobacterium bovis*, and the attenuated form of the bacillus Calmette-Guérin (BCG vaccine). Cutaneous tuberculosis can be exogenous; endogenous: caused by contiguity or autoinoculation and by hematogenous spread; induced by the Calmette-Guérin bacillus and manifest as a tuberculid. The diagnosis of the infection is carried out through the direct test, culture, histopathology, tuberculin skin test, polymerase chain reaction, interferon-gamma release assay, and genotyping. Drugs used comprise isoniazid, rifampicin, pyrazinamide and ethambutol.

## Introduction

Tuberculosis (TB) is an ancient, cosmopolitan infectious disease caused by *Mycobacterium tuberculosis* that occurs with greater incidence in developing countries, constituting one of the most serious public health problems, a constant threat to health, which incapacitates and causes the death of a large number of people. TB is considered by the World Health Organization (WHO) as a worldwide disease with more than 4,000 deaths daily, 10.4 million infected individuals annually, and 1.5 million deaths from this infection every year. With the emergence of the HIV/AIDS pandemic, the disease is the leading cause of morbidity and mortality in patients infected with the Human Immunodeficiency Virus (HIV).^1^, ^2^, ^3^ In 2018, 484,000 cases of rifampicin-resistant tuberculosis were registered worldwide, of which 78% were multidrug-resistant TB (MDR-TB).^1^, ^2^, ^3^, ^4^, ^5^, ^6^, ^7^, ^8^

China, India and Indonesia account for 45% of TB cases worldwide.^1^, ^2^, ^9^ Regarding the Southeast Asia and regions of Africa, its mortality rate represents 85% of all deaths due to tuberculosis.^1^, ^2^, ^9^, ^10^, ^11^

In 2018, the disease caused 4,490 deaths in Brazil. In 2019, 73,864 new cases of TB were registered in Brazil – an incidence of 35.0 cases/100,000 inhabitants. In Brazil, an enormous increase in new cases of TB-HIV coinfection was detected between 2010 and 2018.^3^, ^4^, ^5^ The risk of acquiring TB depends on the coexistence of conditioning factors such as genetic factors, age, sex, malnutrition, alcoholism, smoking status, environmental factors, the immune system status of each individual – the increased use of immunosuppressive drugs for the treatment of autoimmune diseases, neoplasms, transplant recipients – development of MDR-TB, HIV infection, previous pulmonary disease, in addition to other comorbidities.^1^, ^2^, ^3^, ^4^, ^5^, ^6^, ^7^, ^8^, ^9^, ^10^

Studies have reported that the immunosuppressive condition, determined by HIV/AIDS infection, predisposes to the contagion and illness of afected individuals. The chance that infection with the bacillus will develop into tuberculosis in immunocompetent individuals is 10% over one’s lifetime, whereas it may reach 10% each year in those infected with HIV.^12^

Extrapulmonary TB represents 20% of the cases of the disease, which includes pleural TB, osteoarticular TB, genitourinary TB, ocular TB, abdominal TB, breast TB, tuberculous meningitis, and pericarditis, cutaneous tuberculosis, among other affected organs.^9^, ^11^, ^13^, ^14^

Cutaneous tuberculosis (CTB) is a rare form of mycobacteriosis that accounts for 1% to 2% of all forms of extrapulmonary tuberculosis.^9^ The main etiological agent is *Mycobacterium tuberculosis* and occasionally *Mycobacterium bovis* and the bacillus Calmette-Guérin (BCG vaccine, an attenuated strain of *M. bovis*).^3^, ^8^, ^9^, ^10^, ^11^, ^15^

In 1826, Théophile Laënnec (1781–1826), the inventor of the stethoscope (1819), described the first case of CTB in the world that killed Laënnec himself. Robert Koch (1843–1910) identified and isolated *M. tuberculosis* only in 1882.^7^, ^8^, ^9^, ^13^

It is important to emphasize that there are infections that are not caused by the *Mycobacterium tuberculosis* complex and by *Mycobacterium leprae* and *Mycobacterium lepromatosis*, but produced by non-tuberculous or atypical mycobacteria (NTM), among which the following stand out: *Mycobacterium avium* complex (MAC), *Mycobacterium ulcerans*, *Mycobacterium marinum*, *Mycobacterium haemophilum*, *Mycobacterium fortuitum*, *Mycobacterium chelonae*, *Mycobacterium kansasii*, *Mycobacterium abscessus*, *Mycobacterium scrofulaceum*, *Mycobacterium simiae*, *Mycobacterium malmoense*, *Mycobacterium xenopi*.^7^, ^8^, ^9^, ^10^, ^11^, ^13^

CTB is prevalent in young adults, particularly women, tuberculosis verrucosa cutis predominates in males, and erythema induratum of Bazin in females.^8^, ^9^, ^10^, ^11^, ^16^

## Etiology and pathogenesis

*Mycobacterium tuberculosis* was identified and isolated by Robert Koch in 1882. It is an obligate intracellular, aerobic, straight or slightly curved, immobile, non-sporulating pathogen, measuring 1 to 10 µm long and 0.2 to 0.6 µm wide, an acid-fast bacillus (AFB), with the ability to survive and multiply inside macrophages. Its genome consists of approximately 4,000 genes, of which about 200 are responsible for encoding enzymes for fatty-acid metabolism, and another 170 encode families of proteins related to antigenicity, some related to the metabolism of the bacillus and constituents of the cell wall – lipids, proteins, carbohydrates – however, most genes play an important role in the invasion of the immune system.^10^, ^11^, ^13^, ^17^^,^^18^ The high concentration of lipids on the cell wall is responsible for the resistance/survival of the microorganism; nevertheless, it shows sensitivity to heat and ultraviolet radiation.^9^, ^10^, ^11^, ^18^

Tuberculosis research has played an important role in the birth of immunology field of studies; however, the relationship between the two has been ambivalent from the start.^19^

Chen et al. propose that: “proteins are the most important antigens of *M. tuberculosis* and can induce T-cell immune responses and other allergic reactions, including late-onset hyperreactive cell immune responses. The caseous necrosis that occurs in the disease is induced by lipoids.”^9^

The T-cell immune response act in the host defense mechanism against bacteria, dendritic cells, activated macrophages, which phagocytose and destroy the mycobacterium, for antigen presentation to lymphocytes, in which a series of biological events are processed, highlighting the important protective function of CD4+ and CD8+ T lymphocytes, which secrete cytokines to eliminate the bacillus of Koch (BK).^9^, ^13^, ^15^ According to Sehgal et al.^19^ one can consider the spectral concept in cutaneous tuberculosis, based on bacteriological, histopathological and immunological parameters, similarly to what is described in leprosy and pulmonary tuberculosis. At one end, lupus vulgaris, with active cell immunity and apparently normal immunoglobulin, and at the other end, scrofuloderma, with less active cell immunity and increased humoral response, evidenced by elevated serum levels of immunoglobulins and reduced C3.^20^

T-cells are responsible for the polarization of the disease in the host since they can restrain *M. tuberculosis*, developing the paucibacillary form or, through immunological suppression, allow the survival of the pathogen, leading to the multibacillary form. The Th1 and Th17 pathways are the main effectors involved in the pathogenesis of TB, mediating protection against the etiological agent. The regulatory T (Treg) cells subgroup also plays an important role in the process, as it maintains the cell-mediated immunity effector cells in homeostasis.^20^, ^21^, ^22^ The most important component of the defense against TB through the Th1 pathway is the production of the cytokine IFN-γ, which activates macrophages and stimulates phagocytosis, phagosome maturation, production of reactive nitrogen intermediates and antigen presentation. Although IFN-γ is one of the most important cytokines for the protective response against *M. tuberculosis*, it is not enough to control the pathogen.^23^, ^24^, ^25^ The Th17 cells produce IL-17, which plays a major protective role in TB, observed through the survival of neutrophils in the early stages of infections, providing control of the bacterial load.^26^, ^27^, ^28^, ^29^, ^30^

The suppressive mediation of Treg cells occurs mainly through the secretion of the immunosuppressive cytokine TGF-β, which has an anti-inflammatory effect.^31^, ^32^, ^33^ In patients with cutaneous tuberculosis, Treg cells produce a significant amount of TGF-β, which plays an important role in CD4+, CD25+ Treg expansion and also inhibits Th17 cell polarization.^22^

The main target of *M. tuberculosis* in the human species is the lungs; however, all organs and systems of the host can be infected by the microorganism. Most of the infections occur through the airways and 80% of TB clinical forms are pulmonary.^7^, ^8^, ^9^, ^10^, ^11^, ^15^, ^17^ Skin integrity is very effective in preventing BK entry, but solutions of continuity of the mucocutaneous integument will favor infection of the host by the BK and, therefore, cutaneous and/or mucosal tuberculous manifestations. 7, 8, 11, 17

## Clinical manifestations and classification

The clinical manifestations of CTB polymorphism can be explained by factors such as the pathogenicity of the bacterial strain, the host immune status, previous treatment, or local factors, including proximity to lymph nodes.^8^, ^9^, ^10^, ^11^, ^17^

The polymorphous lesions of CTB include papules, nodules, infiltrated plaques, ulcers, gummas, verrucous lesions. Their differential diagnosis is mandatory with other diseases of similar clinical expression. This lesion polymorphism has led to the proposition of several complex and controversial classifications.

The bacterial load is the basis for a proposed classification for the disease, which is analogous to the Ridley and Jopling classification for leprosy and includes:

a) The multibacillary form – low immunity to BK, including tuberculous chancre, tuberculosis orificialis, scrofuloderma, acute miliary tuberculosis, and tuberculous gumma.
b) The paucibacillary form – high immunity to *M. tuberculosis*, including verrucous tuberculosis, lupus vulgaris and tuberculids.

The classification for TB based on more robust criteria – pathogenesis, anatomopathological analysis, immune system, clinical picture – accepted worldwide by those who study mycobacteriosis will be adopted in this work:^7^, ^8^, ^9^, ^11^, ^16^, ^17^

a) Exogenous cutaneous tuberculosis: tuberculous chancre and tuberculosis verrucosa cutis.
b) Endogenous cutaneous tuberculosis:

Contiguity or self-inoculation (scrofuloderma, tuberculosis orificialis and some cases of lupus vulgaris);

Hematogenous spread (lupus vulgaris, gummatous tuberculosis and acute miliary tuberculosis).

c) Cutaneous tuberculosis caused by bacillus of Calmette-Guerin (BCG vaccine); post-vaccination lupus vulgaris.
d) Tuberculids: papulonecrotic tuberculid; lichenoid tuberculid (lichen scrofulosorum)
e) Facultative tuberculids: erythema induratum of Bazin -nodular vasculitis.

## Tuberculous chancre

Tuberculous chancre (TC) or primary inoculation tuberculosis results from the direct penetration of *M. tuberculosis* into the skin and/or mucous membranes through trauma, wounds, preexisting dermatosis, surgical procedures with contaminated material in people who do not have innate or adaptive immunity to *M tuberculosis*. Individuals also acquire the disease through mouth-to-mouth breathing, circumcision, injections with contaminated hypodermic needles, after tattooing procedures, piercings, at insulin application sites. Infection of the oral mucosa by *M. bovis* can occur by ingestion of unpasteurized milk and by dental extraction. It is the most common form in children; however, adolescents and young adults can also be infected in this way.^7^, ^8^, ^9^, ^10^, ^11^, ^13^, ^34^

### Clinical manifestations

TC manifests two to four weeks after infection as a papule, infiltrated plaque or nodule that develops into an ulcerative lesion located more frequently on the face (including the conjunctiva), upper limbs (mostly the hands) and lower limbs. The ulcer is painless, shallow, with a granular background, an infiltrated base that can reach a few centimeters in diameter, and is difficult to heal. After two to eight weeks, lymphangitis and regional lymph node disease ensue, constituting the primary tuberculous complex in the skin, similar to the Ghon complex of the lung.^7^ The lymph nodes may liquefy, ulcerate and eliminate necrotic/necropurulent material. Paronychia resulting from infection of the fingers by the bacillus is not so rare.^7^

### Histopathology

An acute inflammatory infiltrate of neutrophils can be seen at the beginning of the infectious process, followed a few weeks later by granuloma organization, associated with caseous necrosis and the presence of numerous acid fast bacilli (AFB), demonstrated by Fite-Faraco or Ziehl-Neelsen stains.

### Diagnosis

Laboratory confirmation of the etiological agent by direct screening and culture for AFB, PPD (purified protein derivative), histopathological analysis, interferon-gamma release assay (IGRA) and polymerase chain reaction (PCR) for BK.

### Differential diagnosis

It must be established with NTM, syphilis, bartonellosis, sporotrichosis, and leishmaniasis.

## Tuberculosis verrucosa cutis

**Figure 1:**
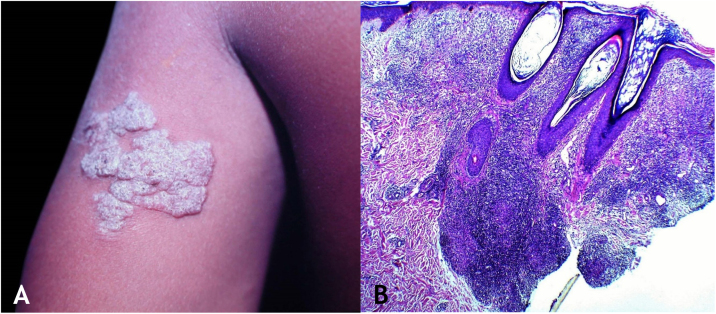
(A), Tuberculosis verrucosa cutis (TVC): verrucous plaque with centrifugal expansion on the left arm. (B), TVC: epidermis with focal hyperplasia and dermis showing granulomatous inflammation involving follicular structures and vessels, (Hematoxylin & eosin, ×40).

Tuberculosis verrucosa cutis results from the exogenous inoculation in individuals previously infected with BK, with high immunity to the microorganism. It is the most common paucibacillary variant in healthcare professionals – physicians, pathologists, veterinarians, biomedical technicians, nurses, autopsy technicians – in farmers, butchers and is unusual in people with other activities (Fig. 1A).^7^, ^8^, ^9^, ^10^, ^11^

### Clinical manifestations

Initially, the lesion is a papule/papulo-pustule with an erythematous halo or a nodule with hyperkeratosis on the surface, developing into a verrucous plaque with centrifugal growth, sharp edges, and the presence of purulent secretion foci. The lesion is usually single, preferably located on the hands, but other areas of the skin may be involved, such as the lower limbs, particularly in children.^7^, ^8^, ^13^, ^16^^,^^17^ This lesion eventually progresses to spontaneous healing with atrophic scarring.

### Histopathology

Hyperkeratotic pseudoepitheliomatous hyperplasia is observed and, a lymphocytic inflammatory infiltrate, neutrophil abscesses in association with epithelioid cell granulomas, Langhans-type multinucleated cells, and caseous necrosis are seen in the dermis. AFB are scarce or difficult to identify (Fig. 1B).

### Diagnosis

Clinical suspicion is confirmed through bacilloscopy, culture, IGRA, PPD, PCR, or histopathology.

### Differential diagnosis

It includes the verrucous syndrome (PLECT) – (Paracoccidioidomycosis, Leishmaniasis, Sporotrichosis, Chromomycosis, Tuberculosis (other forms), NTM, verrucous lesions, carcinoma, keratoacanthoma, bromoderma, iododerma, hypertrophic lichen planus.

## Scrofuloderma

Scrofuloderma or *tuberculosis cutis colliquative* is the most frequent form of TB in developing countries, being prevalent in children and young adults, but it also affects the elderly. Depending on the host’s immune system and other conditioning factors, it can affect the host in both the multibacillary and paucibacillary clinical forms.

### Clinical manifestations

*M. tuberculosis* infection develops in lymph nodes, bones, joints, as tuberculous epididymitis, or orchiepididymitis, and the overlying skin is affected through contiguity by the mycobacteria. The coexistence of pulmonary TB is seen in most cases of scrofuloderma.^7^, ^8^, ^9^, ^11^, ^16^, ^17^

**Figure 2:**
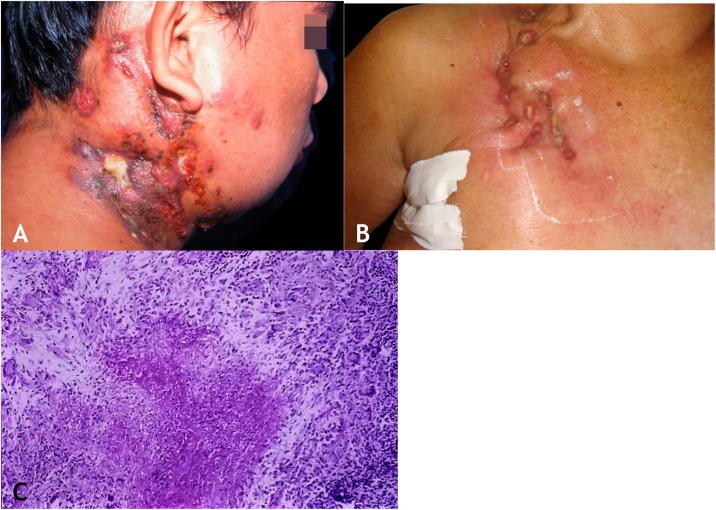
(A), Scrofuloderma – nodules/gummas and ulcerations on the cervical and right mastoid regions associated with lymph node tuberculosis. (B), Scrofuloderma – infiltrated and ulcerated lesions on the right clavicular region. (C), Scrofuloderma – caseation necrosis surrounded by granulomatous inflammation with a palisade of macrophages and Langhans-type multinucleated giant cells, (Hematoxylin & eosin, ×40).

The initial lesion is a subcutaneous nodule – single or multiple – initially firm, which progressively increases in size. After several weeks, it softens, ruptures, resulting in fistulas and ulceration, with the elimination of necrotic or necro-purulent material. They are known as cold abscesses. The cervical, axillary, supraclavicular, and inguinal lymph node chains are most frequently involved in this form of tuberculosis (Figs. 2A and 2B)

### Histopathology

The epidermis is atrophic or ulcerated, depending on the biopsied lesion. The dermis and subcutaneous tissue show caseous necrosis, surrounded by macrophage granulomas, Langhans-type multinucleated giant cells and a small number of lymphocytes. AFB are scarce. In most cases, *M. tuberculosis* is difficult to demonstrate by special stains (Fig. 2C).

### Diagnosis

Clinical findings are complemented by laboratory tests: PPD (strong reactor), culture in specific media, histopathology, IGRA, PCR for BK.

### Differential diagnosis

The following should be considered: paracoccidioidomycosis, actinomycosis, sporotrichosis, syphilitic gumma, NTM, hidradenitis suppurativa.

## Tuberculosis orificalis

Tuberculosis orificalis (TO), also known as acute tuberculous ulcer and ulcerative cutaneous and mucosal tuberculosis, results from self-inoculation of *M. tuberculosis* in the mucosa and periorificial skin in individuals with low immunity and tuberculosis in internal organs – lungs, urogenital, gastrointestinal, in addition to other activity foci. This rare form of TB has a higher incidence in the elderly, who usually have an immunological deficiency and non-reactive PPD.^7^, ^8^, ^9^, ^11^, ^16^, ^17^, ^18^, ^35^, ^36^

Considering the multiple differential diagnoses that genital ulcers raise with other diseases that manifest with this type of lesion - viral, bacterial, fungal infections, pre-cancerous, neoplastic lesions, and other causes - it is essential to carefully investigate for an accurate diagnosis.^37^ Genital tuberculosis occurs more frequently in old, postmenopausal women, almost always related to an active or reactivated tuberculous focus, with a predominance of lesions in the fallopian tubes, in the endometrium, and rarely in the vulvovaginal region. However, the development of a painful ulcer of tuberculous etiology on the left labia majora in a 14-year-old virgin female is an exceptional report in the literature.^37^ The record is a warning to include TO among the proposed diagnoses, even when it appears in adolescents who have not yet started sexual activity.

### Clinical manifestations

**Figure 3:**
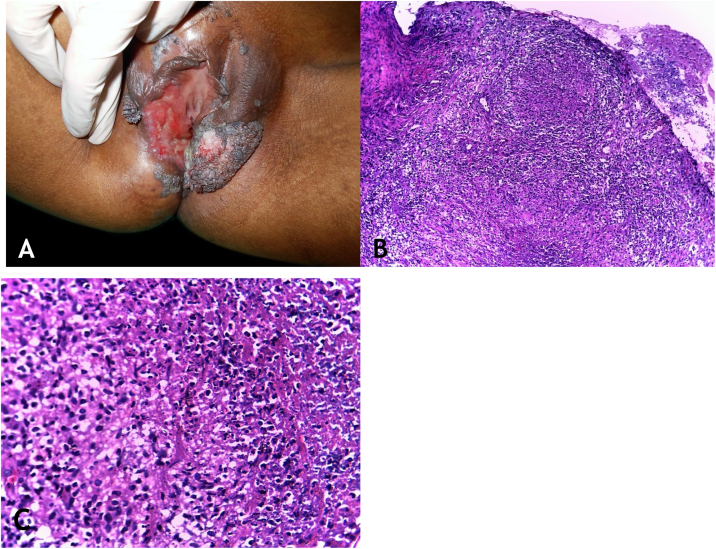
(A), Tuberculosis orificialis (*tuberculosis cutis orificialis*) – Ulcerovegetating lesion affecting the labia majora and minora. (B), TB orificialis – ulcerated epidermis and massive dermal granulomatous inflammatory infiltrate, (Hematoxylin & eosin, ×40). (C), TB orificialis – granuloma associated with caseation necrosis, (Hematoxylin & eosin, ×200). Pictures by: Dr. Maraya Bittencourt.

The sites most often affected by mycobacterioses are the oral cavity, external genitalia, Fallopian tubes, endometrium, anal/perianal regions (Fig. 3A).

Oral cavity lesions can extend contiguously to the pharynx and larynx in the form of very painful ulcerations. The disease starts as a yellowish erythematous papule or nodule, progressing to painful ulceration with irregular edges, infiltrated base, with no tendency to scarring, causing marked oropharyngeal dysphagia.

### Histopathology

Ulceration of the lining epithelium and dermis and granulomas with caseation necrosis — there are numerous AFB, demonstrated by special stains (Figs. 3B and 3C).

### Diagnosis

Clinical aspect of the lesions, complementary exams, investigation of TB in other organs, especially in the lungs.

### Differential diagnosis

Consider recurrent aphthous stomatitis, syphilis, and other sexually transmitted infections with a clinical ulcerative characteristic, deep mycoses, and malignant neoplasms, especially squamous cell carcinoma.^35^

## Lupus vulgaris

Lupus vulgaris (LV) is a type of endogenous, chronic cutaneous tuberculosis, caused by contiguity or self-inoculation (some cases) and by hematogenous or lymphatic spread, in individuals with high or moderate immunity to *M. tuberculosis*, who are tuberculin-positive, with paucibacillary disease, which has a higher prevalence in females than in males, affecting any age group. The infection occurs from a tuberculosis focus of pulmonary, osteoarticular, lymph node origin, or from other organs. It can exceptionally develop after BCG vaccination.^11^, ^12^, ^13^, ^16^, ^18^, ^36^

### Clinical manifestations

LV is manifested by the presence of an erythematous-brownish papule or nodule with a smooth or keratotic surface, a gelatinous consistency that, on diascopy, looks like “apple jelly”.

**Figure 4:**
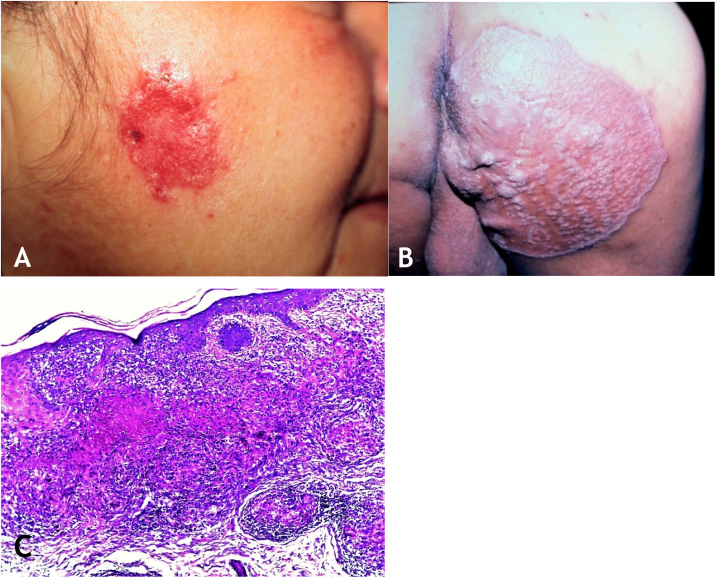
(A), Lupus vulgaris – infiltrated, eroded lesion, with sharp edges, in the right masseteric region. (B), Lupus vulgaris – extensive plaque covered with papules/nodules, scarring areas, with sharp raised edges on the right gluteal region. (C), Lupus vulgaris – the dermis shows granulomatous inflammation with foci of caseous necrosis, (Hematoxylin & eosin, ×40).

The lesion is usually single; however, it can be multiple. Its preferred location is on the face – nose, malar regions, chin, ears – but other regions can be affected, such as the cervical area, upper and lower limbs. The initial lesion progresses to a plaque with centrifugal expansion, with an atrophic center that can ulcerate (Figs. 4A and 4B).

Published case reports with different types of LV lesions have resulted in variants, as follows: psoriasiform, eczematous, annular, ulcerative, vegetating, hypertrophic, and scarring plaques.^9^, ^38^ the ulcerative and vegetating mutilating variants cause severe deformities in the affected sites.^7^, ^8^, ^9^, ^13^, ^17^

The mucosa can be compromised by direct inoculation of *M. tuberculosis* or from the expansion of a skin lesion. malignant degeneration – squamous cell carcinoma, sarcomas, Hodgkin’s lymphoma – has been reported in patients with long-term LV.^7^, ^35^, ^39^

The disease has a chronic course and, if left untreated, will cause deformities due to the destruction of structures in the affected anatomical sites. Spontaneous healing of LV is very difficult, but can occur after several years, leaving an atrophic or deforming scar.

### Histopathology

The epidermis may present with atrophy, acanthosis, pseudoepitheliomatous hyperplasia, or ulceration. In the dermis, tuberculoid granulomas containing Langhans-type multinucleated giant cells and surrounded by lymphocytes can be observed. Caseation necrosis is minor and sometimes absent. AFB is difficult to identify or absent (Fig. 4C).

### Diagnosis

The diagnosis is carried out through the clinical appearance and diascopy of skin lesions, strong reactive PPD, culture in specific media, histopathological analysis, IGRA, PCR for *M. tuberculosis*.

### Differential diagnosis

The following should be included: lupus erythematosus, sarcoidosis, cutaneous pseudolymphoma, leishmaniasis, leprosy, tertiary syphilis, paracoccidioidomycosis, and diseases with similar clinical expression.

## Acute miliary tuberculosis

Acute miliary tuberculosis or disseminated cutaneous tuberculosis occurs by hematogenous spread of the active focus of BK from visceral tuberculosis, commonly from the lungs. The higher prevalence is observed in childhood, in patients immunosuppressed by HIV infection, undergoing chemotherapy, using immunosuppressants for autoimmune disease, and in transplant recipients. It represents a rare form of TB, however, with risk of death for this group of patients. Coinfection with HIV in patients with a CD4 cell count < 100/µL has contributed to the increase in the number of cases of this clinical form of tuberculosis.^8^, ^9^, ^10^, ^11^, ^12^

### Clinical manifestations

The lesions are polymorphic, including papules, vesicles, pustules, nodules, with preferential distribution in the upper and lower limbs, and trunk, but they can be disseminated, usually asymmetric. Whitlow abscesses and ulcerations have been recorded in the literature.^7^, ^8^, ^9^

### Histopathology

The histomorphological picture of the initial lesions shows areas of necrosis and neutrophilic abscesses. As the lesions progress, the presence of histiocytes and the formation of granulomas in the periphery of the infiltrate can be observed. Numerous AFB can be identified in neutrophilic abscesses, as well as vascular thrombi.

### Diagnosis

It is based on the dermatological lesions, general physical examination, bacilloscopy, culture in specific media, anatomopathological analysis, IGRA, PCR, diagnostic imaging.

### Differential diagnosis

The following should be considered: NTM, secondary acneiform syphilis, deep mycoses, sarcoidosis, drug reactions.

## Gummatous tuberculosis

**Figure 5:**
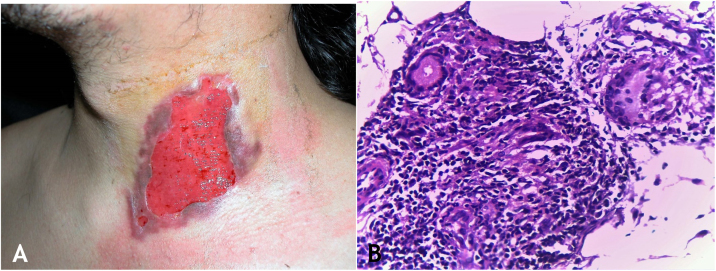
(A), Gummatous TB – irregular ulcer with sharp edges on the cervical region. (B), Gummatous TB – granuloma consisting of lymphocytes, macrophages and multinucleated giant cells, (Hematoxylin & eosin, ×100).

Gummatous tuberculosis (GT) or metastatic tuberculous abscesses is a type of CTB caused by the hematogenous spread of *M. tuberculosis* originating from a primary tuberculous focus.^7^, ^8^, ^9^, ^11^, ^13^, ^18^ GT has a higher incidence in immunosuppressed individuals and in children and adolescents with malnutrition (Figs. 5A and 5B).^7^, ^8^, ^9^, ^17^

### Clinical manifestations

**Figure 6:**
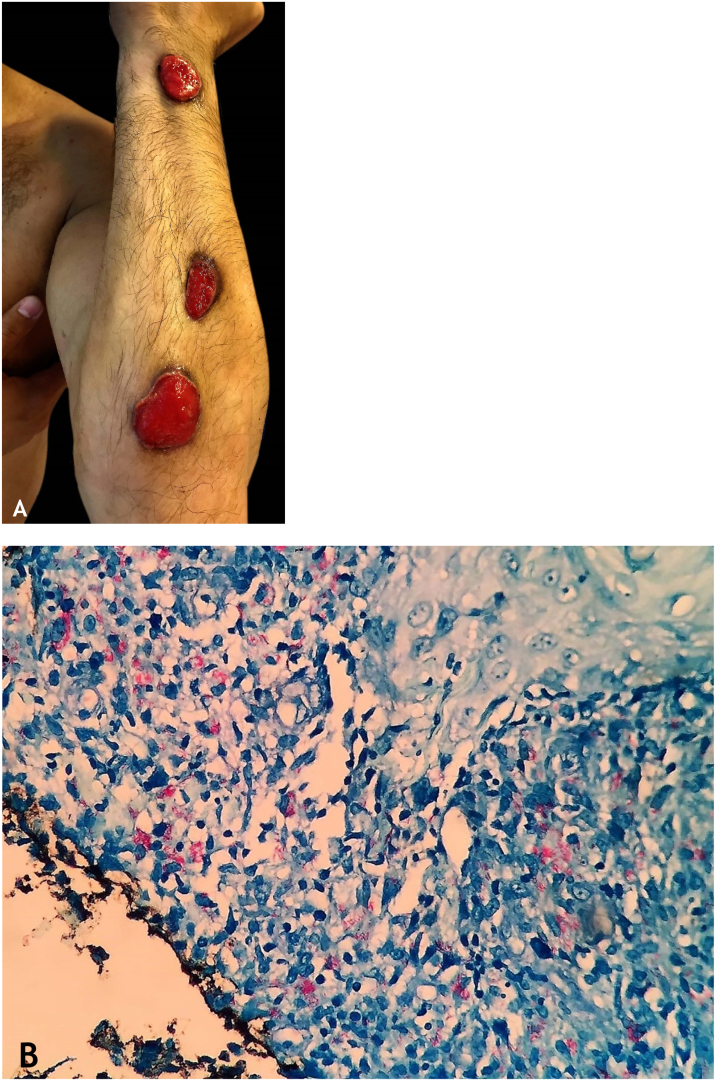
(A), Sporotrichoid gummatous TB – ulcerated lesions along the upper limb. (B), Sporotrichoid gummatous TB - numerous resistant acid-fast bacilli (AFB) in the infiltrate on Fite-Faraco staining. Pictures by: Dr. Deborah Unger.

The initial lesions are firm nodules, progressing to abscesses and later ulceration, draining necrotic material, located on the trunk and on the upper and lower limbs. Some lesions mimic those seen in scrofuloderma. Tuberculous gumma with a sporotrichoid appearance has been the subject of several published studies that emphasize the difficulty in conclusively diagnosing these cases (Fig. 6A).^7^, ^8^, ^9^, ^10^, ^11^, ^16^, ^40^

### Histopathology

AFB is evidenced in areas of massive caseation necrosis. Tuberculous panniculitis cases reveal granulomas with multinucleated giant cells, and AFB are demonstrated by special staining (Fig. 6B).

### Diagnosis

The diagnosis is attained through the clinical aspect of the lesions and laboratory support: bacilloscopy and culture for AFB, anatomopathological analysis, PPD, IGRA, PCR.

### Differential diagnosis

The following should be considered: panniculitis of other etiologies, NTM, deep mycoses (especially sporotrichosis), syphilis.

## Cutaneous tuberculosis after BCG vaccination (Bacillus Calmette-Guerin)

The BCG vaccine was developed by Albert Léon C. Calmette and Jean M. Camille Guérin and used for the first time in 1921. It must be applied after birth until before the age of five, aiming to protect the individual against tuberculosis by preventing the primary infection caused by *M. tuberculosis* from progressing, especially into severe forms of the disease – meningoencephalitis and miliary tuberculosis. Vaccine protection is low in adults.^17^, ^41^ The main hypothesis is that BCG induces effector memory T cells that will protect the individual for 10 to 15 years. Another factor would be that the protective effect of the vaccine would suffer geographic variation.^42^

Approximately 12 vaccines against TB are under development: recombinant BCG (rBCG), *Mycobacterium vaccae*, protein vaccines with adjuvants, viral vector vaccines, DNA vaccines, RNA-based vaccines, and others.^13^, ^42^

**Figure 7:**
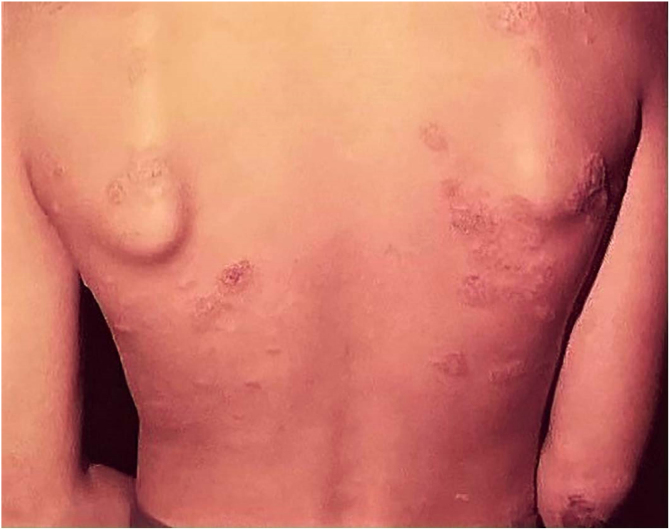
Post-BCG TVC – disseminated annular lesions on the trunk and upper limbs.

Adverse effects occur with any vaccine used in the world, and the BCG vaccine is no exception. The most frequently detected adverse effects of the BCG vaccine include: subcutaneous cold abscess; suppurated regional lymph node disease; ulceration with a diameter > 1 cm; keloid-type scars; Koch’s phenomenon; lupus vulgaris lesions (at or near the vaccine site), scrofuloderma, tuberculid-like lesions; osteoarthritis. Exceptionally, generalized lesions affecting other organs have been recorded (Fig. 7).^41^, ^42^

## Tuberculids

Most recent studies do not consider tuberculids as authentic forms of CTB, but rather a heterogeneous group of clinical entities that result from the host’s hypersensitivity response to *M. tuberculosis*. Bacilloscopy is usually negative for AFB in skin lesions.

The main forms are papulonecrotic tuberculid, lichen scrofulosorum (lichenoid tuberculid), and erythema induratum of Bazin - nodular vasculitis.

## Papulonecrotic tuberculid

Papulonecrotic tuberculid (PNT) is currently a rare form of the disease in industrialized countries; however, it is still common in developing countries with a high prevalence of tuberculosis. Children, adolescents, and young adults are the main victims of this tuberculid variant.^13^, ^17^, ^18^, ^43^

Its association with pulmonary, extrapulmonary, tuberculous mesenteric lymphadenopathy with reactive PPD has been reported.^44^ Evidence of the presence of AFB is difficult to attain or it is absent in skin lesions. However, there are several studies that have identified the presence of *M. tuberculosis* DNA by PCR in 50% of PNT lesions.^7^, ^11^, ^45^ Positive cultures for the microorganism are also reported in skin lesions.^45^ The association of PNT with discoid lupus erythematosus, erythema nodosum, and arthritis have been reported in the literature.^13^, ^43^

### Clinical manifestations

**Figure 8:**
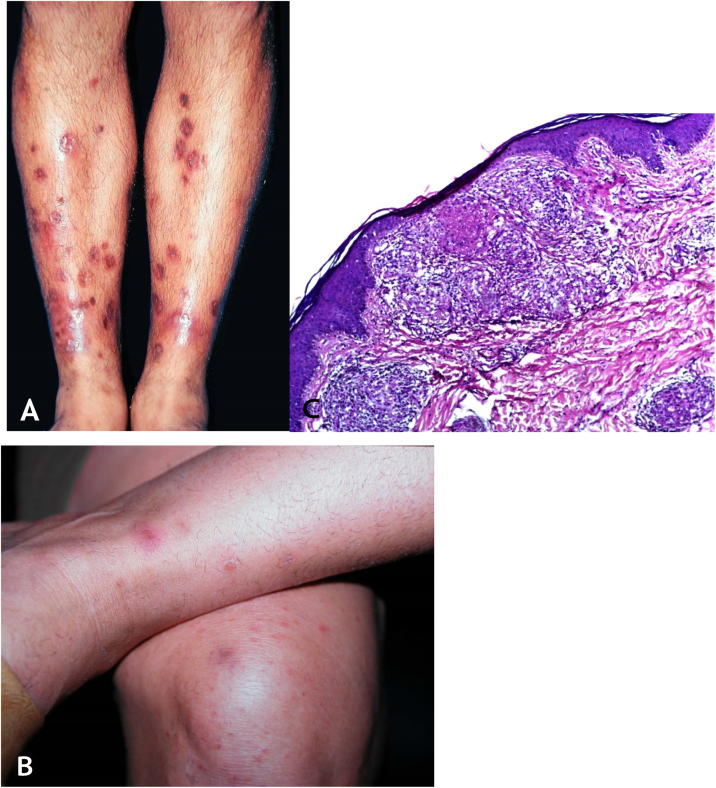
(A), Papulonecrotic tuberculid – “punched-out” lesions on the lower limbs. (B), Papulonecrotic tuberculid – papuloerythematous and papulocrustous lesions in the lower limbs. (C), Papulonecrotic tuberculid – thinning of the epidermis and granulomas with caseation necrosis in the underlying dermis, (Hematoxylin & eosin, ×20).

This is a skin eruption that usually occurs in recurrent outbreaks of erythematous papules that progress with central necrosis and ulceration covered by crusts – called punched-out lesions. The distribution of lesions is symmetric, preferably on the extension surfaces of the upper limbs (including hands), lower limbs, and the trunk. After treatment or spontaneous resolution, varioliform scars are the result (Figs. 8A and 8B).

### Histopathology

Ulcerated epidermis and dermis with an area of wedge-shaped necrosis and dense granulomatous chronic inflammatory infiltrate with few multinucleated giant cells in the surrounding connective tissue. Necrosis may affect hair follicles and granulomatous vasculitis is the alteration detected in some cases (Fig. 8C).

### Diagnosis

The diagnosis is attained through the clinical picture and complementary exams: reactive PPD, histopathological analysis and PCR for AFB.

### Differential diagnosis

The following should be considered: pityriasis lichenoides et varioliformis acuta, leukocytoclastic vasculitis, secondary syphilis, prurigos.

## Lichenoid tuberculid or lichen scrofulosorum

It is characterized by lichenoid papules in individuals highly sensitized to *M. tuberculosis* and/or its proteins. It is frequently associated with tuberculosis in the pleura, lungs, osteoarticular areas, lymph nodes, and other organs. Children and adolescents are the most frequently affected groups, usually from a tuberculous focus.^16^, ^17^, ^36^, ^46^ Some studies show the occurrence of this tuberculid post-vaccination with BCG, as well as infections by NTM, among which the *M. avium*-*intracellulare* complex stands out. However, *M. tuberculosis* DNA has been previously demonstrated by PCR in some cases.^7^, ^14^, ^45^

### Clinical manifestations

Asymptomatic lichenoid papular eruption, with greater concentration on the trunk. There are follicular and/or perifollicular papules, isolated or coalescent, forming plaques with slight scaling on the surface. Spontaneous or treatment-specific resolution may occur.^46^

### Histopathology

The upper dermis reveals a granulomatous inflammatory infiltrate consisting of epithelioid cells, associated with a small number of giant Langhans cells and scarce lymphocytes, affecting hair follicles and sweat glands. In other areas of the dermis, the lymphocytic inflammatory infiltrate is perivascular, with a low density. Absence of *M. tuberculosis* on special staining of the biopsy material and in cultures for AFB. In some studies, the presence of *M. tuberculosis* has been demonstrated by PCR.^45^

### Differential diagnosis

It necessarily includes lichen planus, lichen nitidus, secondary lichenoid syphilis, papular sarcoidosis.

## Erythema induratum of Bazin and nodular vasculitis

Ernest Bazin, a French dermatologist, was the first to describe this form of tuberculid in 1861, which was named after him.^7^, ^8^, ^9^, ^10^, ^11^, ^17^, ^47^

Erythema induratum of Bazin (EIB) and nodular vasculitis (NV) are diseases included in the category of lobular panniculitis, whose clinical manifestations predominate in the lower limbs. The identification of *M. tuberculosis* DNA by PCR in skin lesions in some cases confirms the tuberculous etiology. The term nodular vasculitis or erythema induratum of Whitfield should be reserved for indurated erythema not associated with tuberculosis.^47^, ^48^

### Epidemiology

EIB and NV predominate in young and middle-aged females; however, males can be affected, as well as any ethnic group. Countries with a high incidence of tuberculosis have a higher prevalence of these diseases and venous insufficiency is a frequent association. This variant is very rarely observed in children, although there are several case reports from South Africa and Pakistan. A case of EIB due to BCG vaccination has been recorded.^13^, ^17^

### Pathogenesis

Tuberculosis-associated EIB is observed in populations with a prevalence of mycobacterioses. In patients with a clinical picture of EIB/NV, in which the agent responsible for the skin lesions is not identified through histopathology and culture, it is mandatory to use PCR and specific primers for an accurate diagnosis. It is considered that most cases of EIB are a type IV cell-mediated hypersensitivity reaction, considering that T lymphocytes, macrophages, and Langerhans cells participate in the process. Studies have shown a strongly reactive PPD in these cases and high positivity on the IGRA test for *M. tuberculosis*.

Nodular vasculitis has been associated with several diseases: hepatitis B and C, rheumatoid arthritis, ulcerative colitis, Crohn’s disease, leukemia, hypothyroidism. Some drugs are also associated with NV – propylthiouracil, and etanercept.^13^, ^16^

### Clinical manifestations

**Figure 9:**
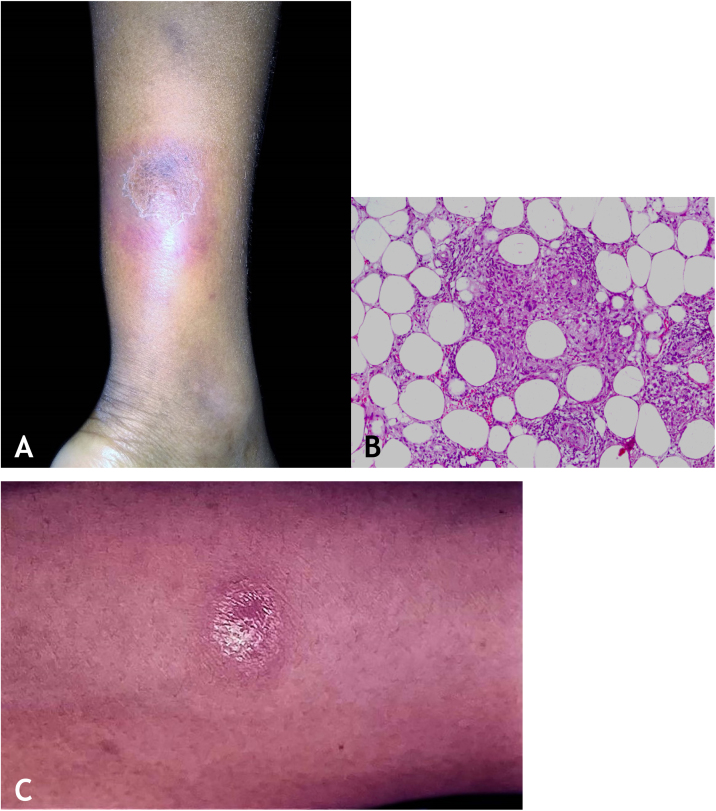
(A), Erythema induratum of Bazin – erythematous-violaceous infiltrated nodule on the posterior aspect of the lower limb. (B), Erythema induratum of Bazin – Lobular granulomatous panniculitis. (C), Erythema induratum of Bazin: Strong reaction to PPD skin test.

They are characterized by erythematous or erythematous-violaceous, painful, isolated, or clustered nodules forming a nodular plaque, which progresses into an ulcer that drains necrotic or necro-purulent material. The preferential location of the lesions is on the posterior surface of the lower limbs (calf); however, other places can be affected: upper limbs, face, gluteal regions, thighs, and feet. The ulcer has sharp, raised edges, an hemorrhagic background, crusts and an infiltrated base. Regression of the lesion with scarring and subsequent recurrence is observed in most cases. Reactive PPD is observed in patients with a tuberculous focus (Fig. 9A).

### Histopathology

An incisional biopsy is recommended to include as much of the adipose tissue as possible, especially to analyze the involvement of the adipose lobe present in the material. Punch biopsies cannot obtain enough adipose tissue for a more accurate histopathological analysis.

Microscopic findings in EIB/NV are lobular or mixed septal/lobular granulomatous panniculitis, with an inflammatory infiltrate consisting of lymphocytes, epithelioid histiocytes, plasmocytes, and Langhans-type multinucleated giant cells. Other identified histological alterations include adiponecrosis, areas of caseation necrosis, and vasculitis compromising adipose tissue arteries and veins. Fibrosis is observed in lesions with a long-term evolution. In general, AFB are not identified on special stains (Fig. 9B).

### Diagnosis

It is based on the clinical manifestations and complementary tests: positive PPD, histopathological analysis, and PCR for *M. tuberculosis* DNA (Fig. 9C).

### Differential diagnosis

It should include erythema nodosum, pancreatic panniculitis, lipodermatosclerosis (sclerosing panniculitis), panniculitis caused by alpha-1 antitrypsin deficiency, other types of vasculitis, especially cutaneous polyarteritis nodosa, sarcoidosis, and subcutaneous Sweet syndrome.

## Diagnosis of cutaneous tuberculosis

*Mycobacterium tuberculosis* direct test or bacilloscopy aims to detect the microorganism in smears of biological material stained by special techniques, with the Ziehl-Neelsen technique being the most often employed in laboratories. There is low positivity for BK, <10%.

Culture on solid media, such as Löwenstein-Jensen and Ogawa-Kudoh of material from lesions shows detection of growth in 14 to 30 days; however, it may be necessary to wait up to eight weeks. CTB lesions show low positivity for mycobacteria (23% of cases).

Culture of BK by the automated and non-radiometric BACTEC MGIT960 system allows detection of mycobacteria within 7 to 15 days. The disadvantage is the high cost of the technique; however, the combination of this technique with the Löwenstein-Jensen medium significantly reduces the time necessary to isolate the mycobacteria and increases the sensitivity in cases of skin lesions.

The Xpert®MTB/RIF test uses molecular techniques with nucleic acid amplification to detect BK and rifampicin-resistant strains. The test increases the sensitivity of the diagnosis.

The tuberculin test is an intradermal inoculation of 0.1 mL, equivalent to 2TU (tuberculin unit) of a purified protein derivative of *M. tuberculosis* (PPD-RT23, in Brazil) on the anterior surface of the left forearm to assess the patient’s cell immune response. The reading is carried out 48 to 72 and up to 96 hours after the application, which is performed with a specific millimeter ruler to measure the largest cross-sectional diameter of the induration. The patient is considered to be infected with BK when the patient has an induration ≥ 5 mm (Fig. 9).

### Interferon-gamma release assay

The IGRA test is used to identify individuals with latent *M. tuberculosis* infection (LTBI); to identify individuals more likely to have LTBI and/or higher risk of becoming ill; to indicate the correct treatment; to notify patients who will be treated for LTBI and to monitor the treatment of these patients.

One should consider that PPD has limitations, including cross-reaction with NTM and with BCG vaccine; false-negative results in immunocompromised patients (e.g., HIV positive); the possibility of error in the test induration measurement; the need for two or three readings after 48–72–96 hours.

The target population of this test is HIV-positive patients, patients with renal failure undergoing hemodialysis, and patients with rheumatoid arthritis and psoriasis on immunobiological therapy.

### Tests available in Brazil: QuantiFERON-TB Gold Plus® (QTF-G) and T-SPOT.TB

The PPD and IGRA tests must be used simultaneously to detect LTBI. Note that these tests cannot differentiate LTBI from active infection.

Polymerase chain reaction (PCR) is a molecular technique that attains amplification of specific sequences of DNA and RNA nucleic acids. It is an important tool in the genotyping of pathogens, in atypical lymphoid infiltrates of T cells, in the investigation of malignant neoplasm translocations, in addition to other clinical entities.

In CTB, the biological material to be analyzed can be fresh tissue or embedded in paraffin. The general sensitivity of PCR found in several studies of clinical forms allows a more accurate diagnosis of the mycobacteriosis.

**Histopathology of CTB** – see detailed descriptions in the clinical forms.

### CTB treatment

The anti-tuberculosis drugs isoniazid, rifamycin, pyrazinamide, and ethambutol are the first-line treatment for all types of CTB, following the standard WHO recommended regimen for the management of new cases of pulmonary TB.^49^ The intensive phase starts and lasts for two months, followed by the maintenance phase for four months.

Doses of drugs used in the intensive phase for adults and adolescents include: rifampicin (450 mg/day for < 50 kg of body weight and 600 mg/day for > 50 kg of body weight); isoniazid (300 mg/day), pyrazinamide (1,500 to 2,000 mg/day); ethambutol (750 mg/day for < 50 kg body weight and 1,000 mg/day for > 50 kg body weight).

Doses for children: rifampicin (10–20 mg/kg daily), isoniazid (10–15 mg/kg daily), and pyrazinamide (30–40 mg/kg daily).

Doses of drugs used in the maintenance phase: rifampicin (600 mg/day for adults and 10 mg/kg daily for children) and isoniazid (600 mg/day for adults and 10 mg/kg daily for children).^36^

Surgical excision may be considered in refractory lesions to correct deformities in recalcitrant cases.^50^

Tuberculid treatment follows the same regimens recommended for true TB. For EIB, a longer period of treatment is recommended, such as the proposed maintenance of isoniazid for up to two years.^50^ Dapsone, potassium iodide, and doxycycline have been reported as adjunctive treatments to treat inflammation in EIB, whereas corticosteroids or tuberculin protein at different dilutions are occasionally used for desensitization.^50^ The treatment of lupus vulgaris with isoniazid alone has resulted in high cure rates.^51^

Some studies have reported an eventual paradoxical skin reaction in patients undergoing regular treatment for TB, particularly in anergic patients treated for miliary TB. Weeks or months after starting the polychemotherapy, purulent swollen skin lesions appear, whose culture for *M. tuberculosis* is generally positive, representing an immunological phenomenon (and not bacterial resistance) that responds well to continued treatment. ^7^

## Immunobiologicals and latent tuberculosis

In immunocompetent individuals, the host’s natural defense system controls the initial infection caused by *M. tuberculosis*; the carrier remains asymptomatic and develops latent tuberculosis. However, in the presence of diseases or medications that lead to immunosuppression, such as immunobiologicals, latent tuberculosis can be reactivated.^52^, ^53^

Significant advances in the understanding of the pathogenesis of immune-mediated diseases allowed the development of new drugs called immunobiologicals, which revolutionized the therapeutic approach to several diseases, such as psoriasis. In particular, the introduction of tumor necrosis factor-alpha inhibitors (anti-TNFα) constituted one of the most revolutionary advances in the treatment of this group of diseases, as they were the first target in treatment. In addition to the observed efficacy, one of the great advantages of these agents is their safety profile in relation to the absence of toxicity in target organs, such as nephro- or hepatotoxicity, characteristic of conventional systemic treatments. However, as the blocking occurs in the initial phase of the immunological cascade, this therapeutic class leads to a broader immune suppression, which is not as selective and, therefore, does not have such a strong immunological safety profile.^54^

TNF-α is a cytokine that plays a central role in the establishment and maintenance of the inflammatory response against infections, being crucial for the formation of granulomas, which represent an essential defense function against intracellular pathogens such as mycobacteria. Anti-TNF-α therapy compromises the physiological immunoinflammatory responses mediated by TNF-α and may alter the formation and maintenance of granulomas, leading to TB reactivation or spread.^55^

In patients receiving anti-TNF-α treatment, the relative risk of TB infection ranges from 1.5% to 17%, being higher with anti-TNF-α monoclonal antibody therapy (adalimumab and infliximab) than with therapy with soluble TNF-α receptor (etanercept).^56^, ^57^

In an attempt to better seek the specific target to be blocked in the treatment of autoimmune diseases, anti-interleukin immunobiologicals emerged, which are more selective and, therefore, have a much better safety profile. It is possible that blocking anti-IL does not carry the same risk of TB reactivation as TNF-α inhibitors.^58^ Th17-related cytokines seem to contribute to immunological protection against primary *M. tuberculosis* infection, and their expression levels decrease in patients with TB.^58^

Despite the effect on the defense mechanism against *M. tuberculosis* infection of IL-17 and IL-23, the available safety data on anti-IL-17 and anti-IL-23 immunobiologicals demonstrate that this effect may be lower than expected, and this may constitute an advantage of this group of immunobiologicals in relation to anti-TNF and anti-IL 12/23. However, further studies on the safety of anti-interleukin immunobiologicals are needed to further elucidate the issue.^59^

It is recommended by international guidelines that prior to treatment with immunobiological drugs, a careful TB investigation should be carried out.^58^ The investigation should be performed with the simultaneous use of PPD and IGRA and chest radiography (PA and profile), performed periodically.^59^

Although PPD is a test to assess personal exposure to TB, in patients with psoriasis there is a possibility of false-positive results due to pathergy. A decreased response to PPD occurs during treatment with methotrexate, making it difficult to interpret the result.^60^

In Brazil, two therapeutic regimens are recommended for the treatment of latent TB: one with isoniazid, at a dose of 300 mg/day for nine months and the other with rifampicin 600 mg/day, for four months.^58^

The members of the National Commission for the Incorporation of Technologies in the Unified Health System (CONITEC), at the 86th meeting (March 5, 2020), decided that “the subject be made available for public consultation, with a preliminary recommendation in favor of the incorporation of rifapentine (Priftin®) into the SUS, to be used together with isoniazid in the 3HP regimen, for the treatment of individuals with latent *Mycobacterium tuberculosis* infection (LTBI).^61^

## Financial support

None declared.

## Authors’ contributions

Arival Cardoso de Brito: Approval of the final version of the manuscript; design and planning of the study; drafting and editing of the manuscript; effective participation in research orientation; intellectual participation in the propaedeutic and/or therapeutic conduct of the studied cases; critical review of the literature; critical review of the manuscript.

Clivia Maria Moraes de Oliveira: Approval of the final version of the manuscript; design and planning of the study; drafting and editing of the manuscript; effective participation in research orientation; intellectual participation in the propaedeutic and/or therapeutic conduct of the studied cases; critical review of the literature; critical review of the manuscript.

Deborah Aben-Athar Unger: Approval of the final version of the manuscript; design and planning of the study; drafting and editing of the manuscript; effective participation in research orientation; intellectual participation in the propaedeutic and/or therapeutic conduct of the studied cases; critical review of the literature; critical review of the manuscript.

Maraya de Jesus Semblano Bittencourt: Approval of the final version of the manuscript; design and planning of the study; drafting and editing of the manuscript; effective participation in research orientation; intellectual participation in the propaedeutic and/or therapeutic conduct of the studied cases; critical review of the literature; critical review of the manuscript.

## Conflicts of interest

None declared.
